# Factors influencing the virological testing of cornea donors

**DOI:** 10.1097/MD.0000000000008561

**Published:** 2017-11-27

**Authors:** Tobias Röck, Robert Beck, Stefan Jürgens, Karl Ulrich Bartz-Schmidt, Matthias Bramkamp, Sebastian Thaler, Daniel Röck

**Affiliations:** a Centre for Ophthalmology; b Institute of Medical Virology, University of Tübingen, Tübingen; c Department of General Medicine, Ruhr-University Bochum, Bochum, Germany.

**Keywords:** cornea, corneal donation, corneal donor procurement, post mortem blood removal, virological testing

## Abstract

To assess the influence of donor, environment, and logistical factors on the results of virological testing of blood samples from cornea donors.

Data from 670 consecutive cornea donors were analyzed retrospectively. Logistic regression analysis was used to assess the influence of different factors on the results of virological testing of blood samples from cornea donors.

The mean annual rate of donors with serology-reactive or not evaluable result was 14.8% (99 of 670) (range 11.9%–16.9%). The cause of donor death by cancer increased the risk of serology-reactive or not evaluable result (*P* = .0300). Prolonged time between death and post mortem blood removal was associated with a higher rate of serology-reactive or not evaluable result (*P* < .0001). Mean monthly temperature including warmer months, differentiating between septic and aseptic donors, sex, and donor age had no significant impact on the results of virological testing of blood samples from cornea donors.

The cause of donor death by cancer and a prolonged time between death and post mortem blood removal seem to be mainly responsible for serology-reactive or not evaluable result of blood samples from cornea donors. The percentage of discarded corneas caused by serology-reactive or not evaluable result may be reduced by shortening the period of time between death and post mortem blood removal.

## 1 Introduction

Human keratoplasty has been performed successfully for over 110 years.^[1,2]^ It is still the most frequent type of transplantation performed in human beings.^[3]^ In most countries, there is an enormous scarcity of corneal tissue.^[4,5]^ There is a growing need for corneal grafts as a result of demographic changes and an aging population. A growing number of surgical procedures, especially endothelial keratoplasty, is a second reason.^[6]^ During the past 2 decades, an increased percentage of corneas has been discarded before transplantation.^[7]^ Different criteria had been adopted, for example, endothelial assessment, donor medical history, and donor serology to improve donor tissue quality.

Corneal grafts can originate from heart-beating brain-dead multiple organ donors, but they mostly come from deceased donors, so called cadaveric donors.^[8]^ Cadaveric corneal donation has become an indispensable basis for corneal grafts. Virological blood testing of deceased cornea donors is required to minimize the risk of infections for the recipient.^[9]^ However, in contrast to multiple organ donors, in most cases, only cadaveric blood samples are available in cornea donors.^[10]^

Padley et al^[11]^ described viral infections transmitted by tissue transplantation. Thus, virological screening of corneal donors is a prerequisite for eye banking. However, test results are a leading cause for discarding corneas.^[12]^ Wilkemeyer et al^[9]^ reported that hemolysis, autolysis, and bacterial contamination may produce significant changes of postmortem blood samples, which may lead to false-positive serological test results, weakened serological sensitivity, and consequently discarding of donor corneas.

Confronted with the scarcity of donor corneas and inspired by the investigation of Wilkemeyer et al,^[9]^ we decided to assess the influence of donor, environment, and logistical factors on the results of virological testing of blood samples from cornea donors.

To the best of our knowledge, a study evaluating the influence of donor, environment, and logistical factors on the results of virological testing of blood samples from cornea donors has never been undertaken before.

## 2 Methods

### 2.1 Eye donors

From January 2009 to December 2014, data of 670 consecutive cornea donors at the University Hospital Tübingen were analyzed retrospectively. No maximum donor age limit was set; the minimum donor age had been 14 years. In 2016, our study group showed that our collective would have lost nearly 14% grafts for transplantation by using a maximum donor age (>79 years).^[12]^ We came to the conclusion that older donors can not generally be excluded from cornea donation due to scarcity of grafts available for keratoplasty in Germany.

Blood removal times up to 24 hours and enucleation times up to 72 hours post mortem were accepted. A detailed medical history of every cornea donor was obtained by interview with the family, the last attending doctor, interview with the donor's family doctor, and review of any hospital medical records.

Potential donors with cause of death by hepatocellular carcinoma which were caused by viral hepatitis infection (hepatitis B or C) were not considered for donation.

Potential donors with high-risk sexual behaviors or intravenous drug use and consequently high-risk individuals for any of the infectious pathogens (human immunodeficiency virus [HIV], hepatitis B virus [HBV], or hepatitis C virus [HCV]) were not eligible to donate.

The consent and medical history had been recorded. Logistic regression analysis was used to assess the influence of different factors like sex, donor age, cause of death, mean monthly temperature, and time between death and post mortem blood removal on the results of virological testing of blood samples from cornea donors.

This study was approved by the institutional review board of the University of Tübingen and adhered to the tenets of the Declaration of Helsinki.

### 2.2 Blood samples

The post mortem blood samples had been collected from the subclavian vessels, femoral vessels, or by direct intracardiac puncture. The skin was disinfected with Softasept N (B. Braun Melsungen AG, Melsungen, Gemany) before blood removal. The procedure was practiced in the order mentioned above until blood had been found.

A sterile 10-mL syringe (B. Braun Melsungen AG, Melsungen, Germany) with a large cannula (0.9 mm × 70 mm) (B. Braun Melsungen AG, Melsungen, Germany) had been used for all types of puncture. All blood-taking methods were performed by an experienced ophthalmic resident. Blood was transferred into a Sarstedt 9.0-mL K3E S-Monovette (Sarstedt, Nümbrecht, Germany) containing 1.6 mg ethylenediaminetetraacetic acid (EDTA)/mL blood for the Institute of Medical Virology (University Hospital of Tübingen).

### 2.3 Virological testing

Samples from cornea donors were transported and stored at +4°C. All samples had been tested immediately at the Institute of Medical Virology (University Hospital of Tübingen). Commercially available tests screening donor serum had been used according to the manufacturers’ instructions. Blood samples had been drawn for mandatory tests of infectious diseases, including antibodies to hepatitis B core antigen (anti-HBc), hepatitis C virus (anti-HCV), HIV antigen, and antibodies (anti-HIV 1/2, p24 antigen), and also for hepatitis B surface antigen (HBs-antigen).

From 2011 to present, blood samples from cornea donors were tested routinely using the ABBOTT Architect i1000/SR system with the assays anti-HBc II, anti-HCV, HIV Ag/Ab Combo, and HBsAg Qualitative II. All tests are chemiluminescent microparticle immunoassays (CMIA; Abbott GmbH & Co. KG, Wiesbaden, Germany). Performance has also been established for the use of cadaveric blood specimen (serum or plasma). Before 2011, the predecessor model Abbott Axsym System was used with microparticle enzyme immunoassay (MEIA).

Donors’ corneas were discarded based on reactive serological testing for at least 1 marker. A serology-reactive result means that the serology is reactive to any of the 3 viruses tested, including hepatitis B, hepatitis C, and HIV. We report false-positives and true-positives. In case of a reactive/positive screening test, the cornea was discarded for reasons of risk avoidance.

The COBAS AmpliPrep/COBAS Amplicor HCV test and the COBAS AmpliPrep/COBAS TaqMan HCV qualitative test were used for detection of HCV-ribonucleic acid (HCV-RNA) by polymerase chain reaction (PCR) in EDTA plasma. Both assays allow automated processing, amplification, and detection of HCV genotypes 1 to 6. Assay performance and interpretation of test results was done according to the instructions of the manufacturer (Roche Diagnostics GmbH, Mannheim, Germany). To exclude false-negative results for HCV-PCR every sample has to be checked for PCR inhibitors by so called internal control. If the internal control has a negative result this is an indication for PCR inhibitors in the sample, and in these cases, the HCV-PCR has to be interpreted as not evaluable.

### 2.4 Evaluation

We collected relevant donor and storage data, for example, donor age, sex, cause of death, and time between death and post mortem blood removal. Mean monthly temperature of Stuttgart-Echterdingen, close to Tübingen, had been obtained from the homepage of the German meteorological service.^[13]^ There is no weather station in Tübingen; therefore we took the data of the next weather station in Stuttgart-Echterdingen. The “distance as the crow flies” is around 20 kilometres.

### 2.5 Statistical analysis

Statistical analysis of the data was conducted using the Statistical Packages for the Social Science (SPSS 18.0). Univariate analyses and logistic regression were used as appropriate. Quantitative variables were expressed as mean ± standard deviation. Odds ratios (ORs) are quoted with 95% confidence intervals (95% CIs). *P* < .05 was considered to be statistically significant.

## 3 Results

**Figure 1:**
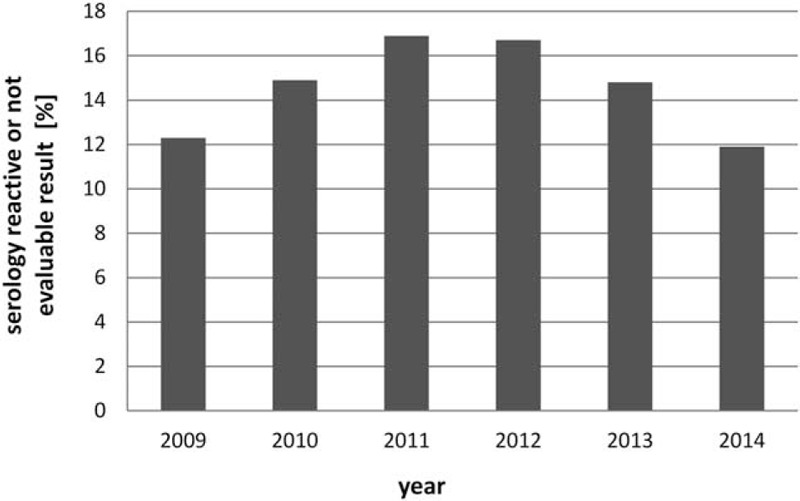
Mean annual rate of donors with serology-reactive or not evaluable result. The mean annual rate of donors with serology reactive or not evaluable result was 14.8% (range 11.9%–16.9%).

This retrospective study included 670 cornea donors. The male-to-female ratio was 61%:39%. Mean donor age was 71 ± 14 years (range 16–93 years). Mean annual rate of donors with serology-reactive or not evaluable result was 14.8% (99 of 670) (range 11.9%–16.9%) (Fig. 1). Mean rate of donors with serology-reactive or not evaluable result was 12.3% in 2009, 14.9% in 2010, 16.9% in 2011, 16.7% in 2012, 14.8% in 2013, and 11.9% in 2014.

Mean time between death and post mortem blood removal had been 12.4 ± 5.8 hours, and mean time in the group of donors with serology-reactive or not evaluable result had been 15.8 ± 3.6 hours. The most common causes of death were cardiovascular diseases (33.4%), cancer (23.0%), and cerebrovascular diseases (17.9%) (Table 1).

**Table 1:**
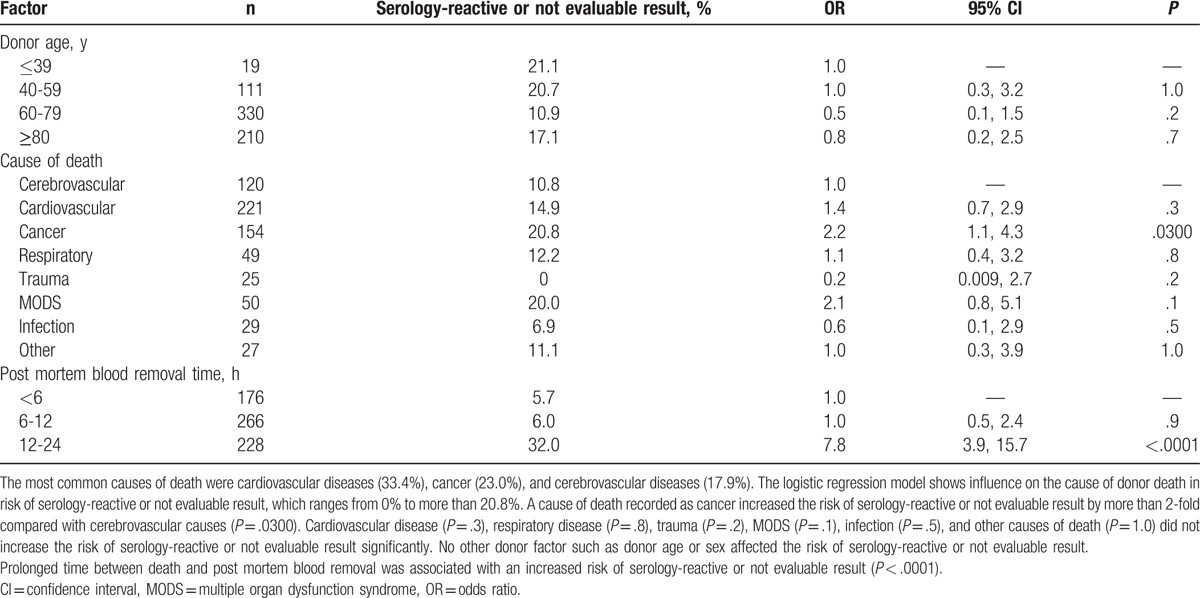
Logistic regression model showing factors influencing the risk of serology-reactive or not evaluable result.

The most common types of cancer were lung cancer (29.2%), followed by bowel cancer (9.1%), breast cancer (7.1%), pancreatic cancer (7.1%), pharyngeal cancer (6.5%), prostate cancer (4.5%), melanoma (3.9%), bladder cancer (3.9%), cholangiocellular carcinoma (3.9%), glioma (3.9%), hepatocellular carcinoma (3.2%), renal cell carcinoma (2.6%), oesophageal cancer (2.6%), cervix cancer (2.6%), laryngeal cancer (2.6%), ovarian cancer (1.9%), gastric cancer (1.9%), uterine cancer (0.6%), testicular cancer (0.6%), trachea cancer (0.6%), liposarcoma (0.6%), and chondrosarcoma (0.6%).

The cause of donor death by cancer was associated with a higher-risk of serology-reactive or not evaluable result (*P* = .0300). These patients have not received any blood transfusions in the last 48 hours before they died.

Cardiovascular disease (*P* = .3), respiratory disease (*P* = .8), trauma (*P* = .2), multiple organ dysfunction syndrome (MODS) (*P* = .1), infection (*P* = .5), and other causes of death (*P* = 1.0) did not increase the risk of serology-reactive or not evaluable result significantly.

Prolonged time between death and post mortem blood removal was also associated with an increased risk of serology-reactive or not evaluable result (*P* < .0001). Sex (*P* = .4), donor age (*P* = .3), and differentiating between septic and aseptic donors (*P* = .3) had no significant influence on the results of virological testing of blood samples from cornea donors.

### 3.1 Risk of serology-reactive or not evaluable result due to seasonal temperature

To explore whether seasonal temperature changes corresponded to the results of virological testing of blood samples from cornea donors, we calculated the mean monthly rate of donors with serology-reactive or not evaluable result and the mean monthly temperature of all years.

The mean rate of donors with serology-reactive or not evaluable result in January was 15.0%, in February 14.5%, in March 19.1%, in April 10.6%, in May 13.2%, in June 13.0%, in July 17.8%, in August 14.3%, in September 15.0%, in October 17.7%, in November 12.5%, and in December 16.1%.

The mean temperature in January was 0.7°C, in February 1.0°C, in March 5.7°C, in April 10.8°C, in May 13.7°C, in June 17.3°C, in July 19.2°C, in August 18.7°C, in September 14.9°C, in October 10.0°C, in November 5.7°C, and in December 2.1°C.

**Figure 2:**
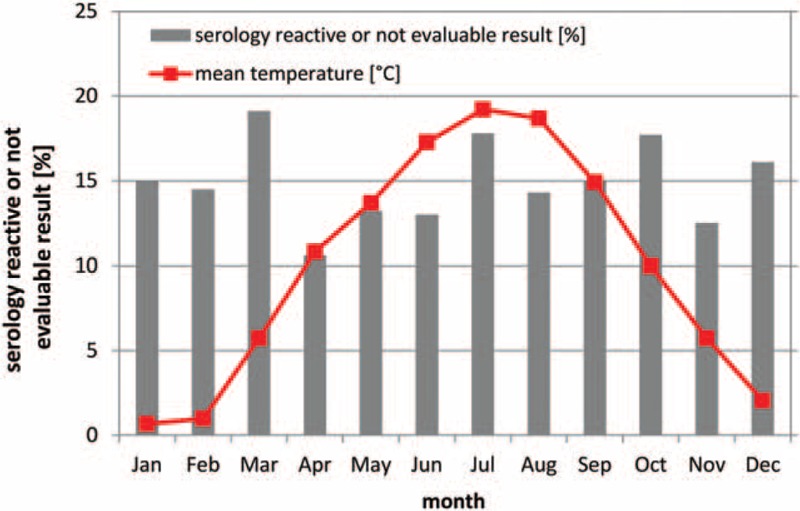
Mean monthly rate of serology-reactive or not evaluable result and mean monthly temperature. Mean monthly temperature including warmer months (May, June, July, August, and September) did not increase the risk of serology-reactive or not evaluable result (*P* = .5).

Mean monthly temperature including warmer months (May, June, July, August, and September) had no impact on the risk of serology-reactive or not evaluable result (*P* = .5) (Fig. 2).

## 4 Discussion

Our investigation illustrates the influence of donor death by cancer or a prolonged time between death and post mortem blood removal on the data of virological testing of blood samples in 670 consecutive cornea donors of Tübingen cornea bank.

It is essential that virological infections of corneal donors are detected. This is essential in protecting recipients of corneal tissue from infections transmitted by tissue transplantation.^[9,11]^ Fornés et al^[14]^ and Heck et al^[15]^ reported that neither the donor history screened by transplant procurement manager nor the plasma or serum testing for virological markers alone is sufficient to ensure tissue safety. Combination of sensitive screening tests and accurate donor history is an essential key to minimize risk of infections of the recipient.

Corneal grafts procurement depends primarily from cadaveric donors and is still an indispensable basis for corneal grafts.^[8]^ Almost all of our grafts are from cadaver donors of the University Hospital in Tübingen and the teaching hospitals with exception of few brain dead organ donors. Premortem blood samples of the donors are often not available. The only way is the use of post mortem blood for serological testing. According to the transplantation law, these samples may not be taken later than 24 hours post mortem.^[16,17]^ Virological screening of corneal donors is a prerequisite for eye banking.

However, virological test results are a leading cause for discarding corneas caused by hemolysis, autolysis, and bacterial contamination.^[9,12,18]^

Warmer temperatures might have had negative impact on donor's blood and consequently results in increased hemolysis. For this reason, we explored whether seasonal temperature changes corresponded to the results of virological testing of blood samples from cornea donors. In our study mean monthly temperature had no impact on the risk of serology-reactive or not evaluable result. Probably, the deceased have been quickly brought into the refrigerator after death.

A prolonged time period between death and blood sample procurement can have negative impact on donor's blood and consequently results in pH changes.^[19]^ These findings seem to be consistent with previous published studies suggesting increased hemolysis by time to sampling after death.^[20]^

Miédougé et al^[21]^ reported a reduced number of indeterminate results of cornea donors by the presence of inhibitors such as hemolysis, heparin, bilirubin, and dextrans. They noted that poor-quality serum samples often generate false-negative or false-reactive results.

False-reactive virological results had been reported for donor samples taken post mortem.^[22]^ Avoiding false-reactive virological results is an important factor in current cornea graft scarcity.^[4,5]^

Our results are in accordance with those of Challine et al^[23]^ and Bensoussan et al^[18]^ who reported a prolonged time period between death and post mortem blood removal seem to be mainly responsible for serology-reactive or not evaluable result of blood samples from cornea donors. For this reason our study group suggests that the percentage of discards caused by serology-reactive or not evaluable result may be primarily reduced by shortening the time period between death and post mortem blood removal.

In contrast to that, Edler et al^[24]^ suggested that infectious serological testing may be extended for blood samples of potential tissue donors collected up to 48 hours post mortem to detect antibodies or antigens for HIV, HBV, and HCV. An expanded time slot would improve the availability of tissue donations significantly. Reinhard^[25]^ also advocated 2011 an extended period between death and post mortem blood removal of cornea donors to avoid the loss of grafts. We are torn between not losing potential corneal grafts and not endangering a recipient with risk of viral infection transmission. For logistical reasons, it is sometimes difficult to get the consent for corneal donation of the relatives within 24 hours. Therefore, we would prefer to extend the time slot between death and post mortem blood removal that no donors are lost. Studies should follow, which investigate whether there exist more false-negative virological results if donor samples were taken after 24 hours post mortem.

According to our results, a possible solution could be shortening the time period between death and post mortem blood removal. Improvement could be arranged by a sufficient staff level including a full-time ophthalmic resident that can react promptly on a potential donor without losing time.^[8]^ Furthermore, potential cornea donor identification is based on a functioning network between intensive care unit colleagues and the responsible cornea bank employees.^[12]^

We perform advancement training twice each year at other clinics, which encourages especially the young and new colleagues, who are just coming from the university, to report a potential donor earlier than without refresher course for cornea donation.

Additionally, over the past few years, a faster reporting system by facsimile (fax) was developed to collect and analyze data on potential cornea donors. Moreover, an employee of the cornea bank can also be reached by telephone 24 hours a day and 7 days a week in the event of a potential corneal donation.

Our primary conclusion suggests that shortening time from death to post mortem blood removal could be a key consideration in receiving meaningful serologic results, and ultimately reducing discards. Another significant determinate in utilization of corneas is the time from death to preservation.^[12]^ In our Cornea Bank, the time from death to blood draw correlates with time from death to preservation.

In our findings the mean annual rate of donors with serology-reactive or not evaluable result was 14.8% (range 11.9%–16.9%), which is comparatively low in comparison with other studies. Challine et al^[23]^ reported that 21.5% of corneas were rejected on the basis of virological test results. The main reason, therefore, could be the different mean times between death and post mortem blood removal. Our time results had been 12.4 ± 5.8 hours and by Challine et al 22.0 ± 12.4 hours. Another reason may be the use of various virological tests.

We found out that the cause of donor death by cancer was significantly associated by a higher risk for serology-reactive or not evaluable result of blood samples from cornea donors. It could be assumed that these patients have received blood transfusions shortly before they died. However, the data showed that these patients have not received any transfusions in the last 48 hours before they died.

A cause could probably be due to the fact that these patients have received a chemotherapy leading to a toxic hemolysis. Jeswani et al^[26]^ in 2015 and Pierce in 2011^[27]^ reported that drug-induced hemolysis and hemolytic anemia are a frequent complication of chemotherapy. It results from interaction of drug with erythrocyte membrane leading to cell lysis.

Nevertheless, some points should be considered before drawing hasty conclusions.

The main limitation of our evaluation is the pilot nature of the observations. Studies in the future will require a larger sample size, which would increase the power of the analysis and the validity of its findings. Other limiting factor of this study is the limited comparability of these data with other publications on the results of virological testing of blood samples from cornea donors. The results may be influenced by the methods of commercially available virological tests screening donor blood samples.

In our opinion, the rate of donors with serology-reactive or not evaluable result may be mainly influenced by a prolonged time period between death and post mortem blood removal, and of cause by the method used for virological testing of blood samples.

## 5 Conclusions

In summary, our investigation illustrates that cause of donor death by cancer and a prolonged time period between death and post mortem blood removal seem to be mainly responsible for serology-reactive or not evaluable result of blood samples from cornea donors. The percentage of discarded corneas caused by serology-reactive or not evaluable result may be reduced by shortening time period between death and post mortem blood removal. Further studies with larger sample sizes are needed to confirm these findings.

## Acknowledgment

We acknowledge support by Deutsche Forschungsgemeinschaft and Open Access Publishing Fund of University of Tübingen.
